# Supplementary material for: Describing the development and implementation of a novel collaborative multidisciplinary approach to deliver foot health supports for individuals experiencing homelessness and its outcomes
Source: PLoS One. 2024 Apr 30;19(4):e0302572. doi: 10.1371/journal.pone.0302572 (PMC11060552; doi:10.1371/journal.pone.0302572)
Supplement: S3 File — (PDF) [file pone.0302572.s003.pdf]

**From:**	Jane Stanley
**To:**	Rajna Ogrin
**Cc:**	Mary-Anne Rushford; Julie Fry; anthonylewis@footscape.com.au; Ian Symmons; Rebecca Mannix
**Subject:**	HEAG 1808 approval - Foot health service linkage for people experiencing homelessness
**Date:**	Wednesday, November 21, 2018 1:36:46 PM
**Attachments:**	image005.png<br>image006.png<br>image007.png<br>image008.png<br>image009.png<br>image018.png

Dear Rajna and team,

Re: HEAG 1808 - *Foot health service linkage for people experiencing homelessness*

Thank you for providing such clear and thorough responses to HEAG members questions about this project. You've done a great job on the PICF!

I note that you will need to get your HREC approval for the changed information. *Can you advise me when this approval is received?*

On the basis that this approval is received, I confirm that cohealth approval is now given for the project, as described in the HEAG application and subsequent clarifications, to proceed in line with the following conditions:

- All research activity undertaken within cohealth must conform to professional and external regulatory standards, including the *National Statement on Ethical Conduct in Human Research*.
- The named researcher is responsible for any personnel associated with the project being made aware of ethics clearance conditions, including research and consent procedures.
- cohealth must be notified of:
    - Any changes to the approved research protocol and project timelines, and
    - Any serious events or adverse and/or unforeseen events that may affect continued ethical acceptability of the project.
- Any publications, presentations, articles and so on that name cohealth must have cohealth approval prior to publication.

Should there be any issues raised with your HREC I'm happy to discuss these with you.

Please do not hesitate to contact me if you have any queries.

Good luck with your project.

Warm regards,

Jane Stanley
Manager: Policy
365 Hoddle Street, Collingwood, VIC 3066
M 0437 051 048
E Jane.Stanley@cohealth.org.au
cohealth.org.au

everyone is welcome at cohealth

cohealth acknowledge the ancestors and traditional custodians of the land on which our offices stand and pay respect to Elders past and present.

From: Rajna Ogrin [mailto:rogrin@boltonclarke.com.au]
Sent: Monday, 12 November 2018 1:45 PM
To: Jane Stanley
Cc: Mary-Anne Rushford; Julie Fry; anthonylewis@footscape.com.au; Ian Symmons; Rebecca Mannix
Subject: RE: Project for ethics review at cohealth - HEAG application 1808

Dear Jane,
Thanks so much for reviewing the project: HEAG application 1808 'Foot health service linkage for people experiencing homelessness'.

Apologies for the delay – we had a hiccup in getting feedback from all the team. Please see attached our response and associated documents.

Please be advised: We will need to get our HREC to approve the changed information and consent form after your group approve a version. I figured if we start with obtaining your approval, I can then ask our HREC for their approval. If there are any issues raised by our HREC, I will need to contact you. Hopefully it will be ok!

If there are any issues with the submitted documents, please do not hesitate to contact me.

Thanks again and we look forward to your response.

Sincerely,
Rajna

Dr Rajna Ogrin PhD BSc BPod(Hons)
Senior Research Fellow
Bolton Clarke Research Institute
Adjunct Associate Professor
Department of International Business and Asian Studies
Griffith University

Adjunct Principal Fellow
Biosignals for Affordable Healthcare
Royal Melbourne Institute of Technology University

Adjunct Research Fellow
Austin Health Department of Medicine
University of Melbourne
Suite 1.01, 973 Nepean Highway, Bentleigh Victoria 3204
Ph 03 8531 2563 **F** 03 8531 2591 **M** 0400 253 459
rogrin@boltonclarke.com.au www.boltonclarke.com.au

Bolton Clarke Research Institute
Better Evidence: Better Health and Wellbeing
