# Supplementary material for: Describing the development and implementation of a novel collaborative multidisciplinary approach to deliver foot health supports for individuals experiencing homelessness and its outcomes
Source: PLoS One. 2024 Apr 30;19(4):e0302572. doi: 10.1371/journal.pone.0302572 (PMC11060552; doi:10.1371/journal.pone.0302572)
Supplement: S2 File — (PDF) [file pone.0302572.s002.pdf]

Bolton Clarke Human Research Ethics Committee

Human Ethics Approval Certificate

NHMRC Registered Committee Number EC00349

Date of issue: 23 July 2018 (supersedes all previously issued certificates)

Dear Dr Ogrin

A Human Research Ethics Committee (HREC) should clearly communicate its decisions about a research proposal to the researcher and the final decision to approve or reject a proposal should be communicated to the researcher in writing. This Approval Certificate serves as your written notice that the proposal has met the requirements of the *National Statement on Ethical Conduct in Human Research* and has been approved on that basis. You are therefore authorised to commence activities as outlined in your proposal application, subject to any specific and standard conditions detailed in this document.

Within this Approval Certificate are:

- Project details
- Participant details
- Conditions of Approval (specific and standard)

Researchers should report to the Bolton Clarke HREC, via the Bolton Clarke HREC Chair, events that might affect continued ethical acceptability of the project, including, but not limited to:

- serious or unexpected adverse effects on participants,
- proposed significant changes in the conduct, the participant profile or the risks of the proposed research, and
- unforeseen events that might affect continued ethical acceptability of the project.

Further information regarding your ongoing obligations regarding human based research can be found via the Bolton Clarke website http://www.boltonclarke.com.au/research/research-ethics or by contacting the HREC Secretariat on 03 9814 2736 or ethics@boltonclarke.com.au

If any details within this Approval Certificate are incorrect please advise the HREC Secretariat within 10 days of receipt of this certificate.

PROJECT DETAILS

**Approved From:**	23/07/2018	**Approved Until:**	31/12/2020 (subject to 6 monthly reports to Bolton Clarke HREC)
**Approval Number:**	170011		
**Project Title:**	Foot health service linkage for people experiencing homelessness		
**Project No:**	193		
**Project Summary:**	The Homeless Persons Program (HPP) are working with key stakeholders at cohealth and the charity Footscape to identify people experiencing homelessness and refer them to cohealth podiatry outreach, with Footscape providing footwear, socks and foot first aid kids as needed. The project's aim is to prospectively audit those people who are in contact with HPP and referred to cohealth and Footscape resources to identify who is accessing these resources and services, their needs and outcomes, to ascertain the population serviced, who is missing out, their needs and what can be done to improve what is being provided.		

INVESTIGATOR DETAILS

Chief Investigator: Dr Rajna Ogrin, Senior Research Fellow, Bolton Clarke Research Institute

Other Researchers:

**Name**	**Affiliation**	**Role**
Anthony Lewis	Footscape	Associate/Assistant/Sub-/Co-Investigator/Researcher
Rebecca Mannix	cohealth	Associate/Assistant/Sub-/Co-Investigator/Researcher

Other Researchers (cont):

Mary-Anne Rushford	Bolton Clarke	Manager, HPP
Julie Fry	Bolton Clarke	Team Co-ordinator, HPP

PARTICIPANT DETAILS

Participants:
Bolton Clarke HPP clients

CONDITIONS OF APPROVAL

Specific Conditions of Approval:
Nil

Standard Conditions of Approval:
The standard conditions of approval require the research team to:

1. Conduct the project in accordance with the Bolton Clarke Guidelines for Researchers Seeking Ethics Approval for Research Projects and the provisions of any relevant State/Territory or Commonwealth regulations or legislation.
2. Include in participant information a Withdraw without prejudice clause.
3. Respond to the requests and instructions of the Bolton Clarke Human Research Ethics Committee (HREC).
4. Advise the Bolton Clarke HREC Chair immediately if any complaints are made or expressions of concern are raised in relation to the project.
5. Suspend or modify the project if the risks to participants are found to be disproportionate to the benefits and immediately advise the Bolton Clarke Bolton Clarke Chair of this action.
6. Stop any involvement of any participant if continuation of the research may be harmful to that person and immediately advise the Bolton Clarke HREC Chair of this action.
7. Advise the Bolton Clarke HREC Chair of any unforeseen development or events that might affect the continued ethical acceptability of the project.
8. Notify the Bolton Clarke HREC if a decision is taken to end the study prior to the expected date of completion or failure to commence the study within 12 months of the HREC approval date.
9. Provide a report on the progress of the approved project every 12 months, from the date of approval for the duration of the project, or at intervals determined by the Committee. Template available on Bolton Clarke website at http://www.boltonclarke.com.au/research/research-ethics/
10. Upon completion of the project, provide a final report. Template available on Bolton Clarke website at http://www.boltonclarke.com.au/research/research-ethics/
11. (Where the research is publicly or privately funded) publish the results of the project in such a way to permit scrutiny and contribute to public knowledge.
12. Ensure that the results of the research are made available to participants.

Modifying your Ethical Clearance:
Requests for variations must be made in writing to the Bolton Clarke HREC Chair. Major changes, depending upon the nature of your request, may require submission of a new application.
