# Supplementary material for: Describing the development and implementation of a novel collaborative multidisciplinary approach to deliver foot health supports for individuals experiencing homelessness and its outcomes
Source: PLoS One. 2024 Apr 30;19(4):e0302572. doi: 10.1371/journal.pone.0302572 (PMC11060552; doi:10.1371/journal.pone.0302572)
Supplement: Attachment — Submitted filename: Response to Reviewers.docx [file pone.0302572.s005.docx]

**Response to reviewers**

Re: Describing the development and implementation of a novel collaborative multidisciplinary approach to deliver foot health supports for individuals experiencing homelessness and its outcomes.

Thank you very much for your very positive responses to our manuscript. Please see below the authors responses to the editorial requirements, followed by responses to the two reviewers:

Table 1. Editorial requirements

| **Editor requirements** | **Authors responses** |
| --- | --- |
| Please ensure your manuscript meets PLOS ONE’s style requirements, including file naming | Thank you. We have complied with your requirements. |
| In this instance it seems there may be acceptable restrictions in place that prevent the public sharing of your minimal data. However, in line with our goal of ensuring long-term data availability to all interested researchers, PLOS’ Data Policy states that authors cannot be the sole named individuals responsible for ensuring data access (<http://journals.plos.org/plosone/s/data-availability#loc-acceptable-data-sharing-methods>).  Data requests to a non-author institutional point of contact, such as a data access or ethics committee, helps guarantee long term stability and availability of data. Please provide non-author contact information (phone/email/hyperlink) for a data access committee, ethics committee, or other institutional body to which data requests may be sent | Thank you for agreeing that the restrictions regarding public data sharing are acceptable for this research. We have included our Human Research Ethics Committee at Bolton Clarke email as the access point to comply with your requirements:  [ethics@boltonclarke.com.au](mailto:ethics@boltonclarke.com.au) |
| Please include captions for your Supporting Information files at the end of your manuscript, and update any in-text citations to match accordingly. Please see our Supporting Information guidelines for more information: <http://journals.plos.org/plosone/s/supporting-information>. | Thank you, we have included this information as directed – including the StaRI checklist as a supplemental. |
| Please review your reference list to ensure that it is complete and correct. If you have cited papers that have been retracted, please include the rationale for doing so in the manuscript text, or remove these references and replace them with relevant current references. Any changes to the reference list should be mentioned in the rebuttal letter that accompanies your revised manuscript. | Thank you – we believe our referencing is complete and correct. We have not retracted any papers. We have added a reference – and have included this in our responses below. |

| **Reviewer responses** | **Authors response** |
| --- | --- |
| Reviewer 1 |  |
| Kudos to the authors for a very commendable program within an underserved population. This manuscript makes for an unconventional and interesting read. | Thanks so much – it is refreshing to receive such positive feedback from a reviewer. We really appreciate it. |
| Will be good for the authors to comment on the funding sources to the program (if any) and long-term sustainability. | The Happy Feet program itself was developed to be incorporated within the existing HPP, community health service podiatry and Footscape programs – as it is part of the core business of all participating groups. As such, it is still occurring. The funding provided by Igniting Change obliged the three parties to formalise the arrangement. This arrangement, now embedded, is continuing.  We have added the following on page 23, line 344 -348 in the section on sustainability:  “As each collaborating organisation provides services to individuals experiencing homelessness as part of their core business, the Happy Feet program enabled the organisational processes necessary to support identification of foot issues by nurses, referral to podiatry as well as sharing the foot health resources to be developed.” |
| The study participants will likely require as much, if not more social services support than foot health services. Would the authors be able to discuss on the socio-economical determinants of health? | We completely agree. It is outside the scope of this manuscript to provide sufficient information to do justice to the topic of socio-economical determinants of health and homelessness. We touched on predisposing factors in the results, and have added a sentences in the discussion on page 25 line 388:  “There are many social determinants of health that influence health outcomes, such as the conditions in which people are born, grow, live work and age, with structural drivers of inequitable distribution of power, money and resources (34).”  Please note, we have added a reference:  World Health Organisation. Closing the gap in a generation: Health equity through action on the social determinants of health. Geneva; 2008. |
| Homeless person need to be empowered, engage in such a program. | This program was funded for a short period, thereby there was insufficient time and resources to enable the engagement and participation of people experiencing homelessness. |

Reviewer 2

| **Reviewer** | **Authors response** |
| --- | --- |
| Publicly Sharing Data policy – needing to add access to data | Please see response to editor above. |
| Excellent project. I congratulate the authors on this new data for the clinical and research community. | Thank you. |
| I am trained in implementation science, and a researcher in limb preservation. My comments and suggestions are intended to be constructive. | Thank you – we appreciate this. |
| Title: why multidisciplinary and not interdisciplinary? | Definitions  Multidisciplinary: draws on knowledge from different disciplines but the boundaries of the disciplines involved are maintained.  Interdisciplinary: involves the linking or combination of two or more disciplines, creating a synthesised whole.  Each collaborator drew on the different disciplines, but worked within their own discipline boundaries making it multidisciplinary. |
| Homeless person need to be empowered, engage in such a program. | Yes – we do agree. The nurses in the Homeless Persons Program are highly trained to work with this group to support their engagement in services. More needs to be done to promote empowerment. |
| Why not use co-design, to ensure that the program corresponds as closely as possible to the needs of homeless people? | This project would have benefited considerably, had we incorporated codesign. Unfortunately we had limited time and resources to enable us to do this. |
| Discussion : It would be interesting to hear about the possibility of scaling this type of program in the future, given that this seems to be a pilot implementation. | This program is very place-based. The collaborating organisations all have the same core client base with the same goals of care, and are all located within easy reach of the CBD, making the program possible.  Unfortunately, other regions don’t have the same resources in their local areas, limiting the ability to upscale to other regions. Particularly podiatry with expertise in working with people experiencing homeless is not widely available. Developing services able to provide the necessary supports would be required.  We have added the following summarising the above on page 24, line 372:  “Of note, there are few podiatry services in operation with expertise to build relationships and trust with people experiencing homelessness, limiting expansion of this type of collaboration to areas in which these services are in operation.” |
